# Changes in Adrenal Function and Insufficiency Symptoms After Cessation of Prednisolone

**DOI:** 10.1001/jamanetworkopen.2025.1029

**Published:** 2025-03-18

**Authors:** Simon Bøggild Hansen, Anja Fenger Dreyer, Nanna Thurmann Jørgensen, Hajir Al-Jorani, Lise Sofie Bislev, Victor Brun Boesen, Stina Willemoes Borresen, Louise Lehmann Christensen, Dorte Glintborg, Ellen Margrethe Hauge, Merete Lund Hetland, Richard Christian Jensen, Søren Andreas Just, Kresten Krarup Keller, Marianne Klose, Kristina Laugesen, Henning Locht, Marie Louise Lund, Jelena Stankovic, Paul M. Stewart, Randi Maria Hanghøj Tei, Anne Voss, Ulla Feldt-Rasmussen, Jens Otto L. Jørgensen, Marianne Skovsager Andersen

**Affiliations:** 1 Aarhus University Hospital, Department of Endocrinology and Internal Medicine, Aarhus, Denmark; 2 Aarhus University, Department of Clinical Medicine, Aarhus, Denmark; 3 Odense University Hospital, Department of Endocrinology, Odense, Denmark,; 4 University of Southern Denmark, Research Unit OPEN, Department of Clinical Research, Odense, Denmark; 5 University of Copenhagen, Department of Clinical Medicine, Faculty of Health and Clinical Sciences, Copenhagen, Denmark; 6 Copenhagen University Hospital, Rigshospitalet, Department of Nephrology and Endocrinology, Copenhagen, Denmark; 7 Aarhus University Hospital, Department of Rheumatology, Aarhus, Denmark; 8 Copenhagen Center for Arthritis Research (COPECARE), Center for Rheumatology and Spine Diseases, Centre for Head and Orthopaedics, Rigshospitalet, Glostrup, Denmark; 9 Svendborg Hospital–Odense University Hospital, Department of Medicine, Section of Rheumatology, Svendborg, Denmark; 10 Aarhus University Hospital, Department of Clinical Epidemiology, Aarhus, Denmark; 11 Aarhus University Hospital, Department of Clinical Biochemistry, Aarhus, Denmark; 12 University of Leeds, Faculty of Medicine and Health, Leeds, United Kingdom; 13 Odense University Hospital, Department of Rheumatology, Odense, Denmark; 14 Randers Hospital, Department of Internal Medicine, Randers, Denmark

## Abstract

**Importance:**

The widespread use of glucocorticoid (GC) therapy may result in GC-induced adrenal insufficiency (GIAI), but the prevalence and clinical implications remain uncertain.

**Objective:**

To ascertain the prevalence and symptoms of GIAI.

**Design, Setting, and Participants:**

Cross-sectional multicenter study at 3 Danish hospitals. Baseline data were collected March 2021 to March 2024 from an ongoing randomized clinical trial. Participants were patients with polymyalgia rheumatica and/or giant cell arteritis who were investigated a median (IQR) of 39 (25-62) days after planned cessation of prednisolone treatment.

**Exposure:**

Prednisolone treatment a median (IQR) of 13 (10-20) months in duration.

**Main outcomes and measures:**

Primary outcome GIAI was defined as a stimulated plasma cortisol level less than 420 nmol/L in response to a short 250 μg corticotropin test (SST). Secondary outcomes were adrenal insufficiency symptoms assessed by the Addison disease-specific quality of life questionnaire (AddiQoL-30), body composition, and muscle function.

**Results:**

Of 267 patients included (145 female [55%]; median [IQR] age 73 [68-78] years), 5 (1.9%; 95% CI, 0.8%-4.3%) had GIAI, whereas 75 (34%; 95% CI, 28%-41%) had symptoms compatible with adrenal insufficiency defined by an AddiQoL-30 score 85 or lower (symptomatic group). The symptomatic group had lower basal cortisol levels compared with the asymptomatic group (263 nmol/L; 95% CI, 242-283 nmol/L vs 309 nmol/L; 95% CI, 295-324 nmol/L; *P* < .001). Factors associated with a low AddiQoL-30 score included female sex (prevalence ratio [PR], 1.68; 95% CI, 1.13-2.51), increased body fat percentage (PR, 2.33; 95% CI, 1.21-4.50), reduced handgrip strength (PR, 2.71; 95% CI, 1.44-5.10) and low Short Physical Performance Battery score (PR, 2.78; 95% CI, 1.42-5.42).

**Conclusions and Relevance:**

This cross-sectional study of 267 patients with polymyalgia rheumatica or giant cell arteritis found a GIAI prevalence of 1.9% after cessation of prednisolone. This is much lower than previously reported and speaks against routine screening, which should be restricted to patients with overt symptoms. The high prevalence of symptoms of adrenal insufficiency in association with lower basal cortisol levels substantiate the clinical challenges of steroid withdrawal and merit future research.

## Key Points

**Question:**

What are the prevalence and symptoms of glucocorticoid-induced adrenal insufficiency after planned cessation of prednisolone treatment?

**Findings:**

In this cross-sectional study of 267 patients with polymyalgia rheumatica or giant cell arteritis, only 5 patients (1.9%) exhibited adrenal insufficiency after prednisolone treatment as judged by the short corticotropin test (SST). However, 34% reported symptoms that could be interpreted as attributable to adrenal insufficiency.

**Meaning:**

These findings suggest that routine testing of adrenal function with an SST after prednisolone treatment is not recommendable, but data support a steroid withdrawal syndrome that is not captured by biochemical testing.

## Teaser

This cross-sectional study evaluates the prevalence and symptoms of glucocorticoid-induced adrenal insufficiency among patients in Denmark treated with prednisone.

## Introduction

Glucocorticoid (GC) replacement therapy has proved lifesaving for patients with adrenal insufficiency (AI) in general, and Addison’s disease in particular, but GC overproduction as seen in Cushing syndrome also carries a poor prognosis if left untreated.^1,2,3^ A common denominator of these conditions in their classical form is their rarity, with annual incidences less than 5 per million.^4,5^ On the other hand, pharmacological GC treatment is very common, illustrated by prescriptions redeemed by 1 to 3% of the population annually, and it is associated with severe adverse effects resembling endogenous Cushing syndrome.^6,7,8,9^ GC treatment also carries a risk of GC-induced AI (GIAI) due to feedback suppression of the hypothalamic-pituitary-adrenal axis.^10,11,12^

The reported prevalence of GIAI after planned cessation of GC treatment, as determined by the short corticotropin test (SST), ranges between 4% to 52% depending on GC dose, route, duration of treatment, and time since cessation, as well as analysis method.^10^ For oral use, the reported prevalence is 49%; however, most data derive from uncontrolled and retrospective audits prone to selection bias.^10^ The majority of studies included were small (less than 50 patients), and nearly all patients were tested 1 day or less after pausing GC treatment and usually shortly after reaching a dose of 5 mg/d. Moreover, very few studies performed liquid chromatography–tandem mass spectrometry (LC-MS/MS) to measure cortisol, which is the criterion standard method due to its superior analytical specificity.^13^

Tapering of pharmacological GC therapy frequently gives rise to GC withdrawal syndrome, the pathophysiology of which is unexplained but consists of physical or psychological dependence to pharmacological GC doses with or without suppression of the hypothalamic-pituitary-adrenal (HPA)–axis depending on the GC dose.^14,15,16^ Despite this apparent dissociation between symptoms of GIAI and biochemically assessed adrenal function, the subject has hitherto not been investigated systematically that we know of.

We therefore aimed to ascertain the prevalence of GIAI and to determine the association between biochemical test outcomes and symptoms in an unbiased manner. In an ongoing prospective multicenter study, we recruited patients with polymyalgia rheumatica (PMR) and/or giant cell arteritis (GCA) shortly after planned cessation of long-term prednisolone treatment. Both disorders are treated in accordance with the European guidelines^17,18^ with starting doses of 40 to 60 mg (GCA) or 12.5 to 25 mg (PMR) tapering over 44 to 53 weeks. When reaching 5 mg prednisolone per day, the dose is reduced by 1.25 mg every 5 weeks until cessation. This strategy is modified according to disease presentation, response, relapse, and adverse effects. All patients underwent an SST and completed questionnaires of AI symptoms, as well as other quality of life (QoL) questionnaires.

## Methods

### Trial Design

This cross-sectional study originates from the Hydrocortisone and Placebo in Patients With Symptoms of Adrenal Insufficiency After Cessation of Glucocorticoid Treatment (REPLACE) protocol, a multicenter, double-blinded, randomized, placebo-controlled 16-week clinical trial (clinicaltrials.gov NCT05193396; EudraCT: 2020-006121-65). The trial compared the outcomes of hydrocortisone and placebo in patients with GCA or PMR with patient-reported symptoms of AI after cessation of glucocorticoid treatment according to the Addison disease-specific quality of life questionnaire (AddiQoL-30) score. The data presented here derive from the screening and recruitment phase of the trial. The Regional Committees on Health Research Ethics for Southern Denmark and the Danish Medical Agency approved the protocol, which was conducted according to good clinical practice. Participants provided written informed consent. The study adheres to the Strengthening the Reporting of Observational Studies in Epidemiology (STROBE) reporting guideline.

### Participants

Individuals aged 50 years or older with PMR or GCA treated with prednisolone for a minimum of 12 weeks were eligible after planned treatment cessation within 2 to 12 weeks before inclusion. Participants were consecutively recruited from patients treated at hospitals in 3 Danish regions (Central, Southern, and Capital). Potential participants were invited by a letter and a subsequent phone call to attend a screening visit. Additionally, participants were invited via advertisement on the official Facebook sites of Aarhus University Hospital and Odense University Hospital.

The major exclusion criteria consisted of severe comorbidity, known disease of the hypothalamic-pituitary-adrenal axis, or use of drugs that interfere with cortisol metabolism or measurement (for more details, see eTable 1 in Supplement 1). Participants were treated at a hospital department of rheumatology or general practitioner. Participants with C-reactive protein levels above the upper reference range at the screening visit were consulted with a dedicated rheumatologist before being invited to a baseline visit. Participants not deemed in remission for their PMR or GCA were excluded.

### Corticotropin Test and Cortisol Measurements

The participants attended a screening visit at any time during the daytime, where an SST was performed. The participant rested 15 minutes in a seated position before a basal blood sample was drawn. Subsequently, 1 ml Synacthen (0.25 mg/ml, CD Pharma) was injected followed by a second blood sample after exactly 30 minutes. Serum/plasma cortisol was measured by LC-MS/MS (148 patients) or immunoassay (Roche Elecsys Cortisol II) (119 patients). The Roche assay displays a high agreement with LC-MS/MS with a correlation coefficient of 0.99.^19^ We defined AI as a stimulated 30-minute plasma cortisol less than 420 nmol/L (to convert to micrograms per liter, divide by 27.588).

### Questionnaires

Participants completed electronic versions of the following questionnaires: AddiQoL-30, CushingQoL, and Single Item Sleep Quality Scale (SQS). AddiQoL-30 is a disease-specific questionnaire developed and validated for patients with primary AI.^20^ The 30 items focus on common symptoms of AI with response categories on a scale from 1 (none of the time/strongly disagree) to 6 (all of the time/strongly agree). Each item yields a score between 1 to 4 arbitrary units, with higher scores indicative of higher QoL, which adds up to a total score ranging from 30 to 120. The items cover 4 dimensions: fatigue, symptoms, emotions, and miscellaneous. We defined clinically significant symptoms of AI as an AddiQoL-30 score of 85 or lower since the mean score in patients with endogenous AI is 85, whereas a healthy random population sample exhibited a mean score of 97 (eTable 2 in Supplement 1).^20^ We subdivided our population into 2 groups according to AddiQoL-30 scores: 85 or lower (symptomatic group) and above 85 (asymptomatic group).

CushingQoL is a disease-specific questionnaire devised for patients with endogenous cortisol excess.^21^ The 12 items cover mental and physical adverse effects of GC excess, and scoring is normalized to a scale from 0 to 100 with lower scores indicating poor QoL. SQS is a single item questionnaire validated against more exhaustive instruments consisting of a 10-point numerical rating scale reflecting the overall sleep quality over the last 7 nights.^22^

### Baseline Visit

As per protocol, the first subset of 129 participants underwent a separate baseline visit, including a detailed interview focusing on prednisolone treatment and medical history in addition to blood sampling and a whole-body dual-energy x-ray absorptiometry scan (Hologic Horizon or Norland). Handgrip strength of the dominant arm was measured 3 times by a dynamometer (Jamar Digital Plus) where the maximal strength (kilogram) was used. Furthermore, the Short Physical Performance Battery was performed, which consists of 3 separate tests, each scored from 0 to 4 points yielding a total score between 0 (worst performance) and 12 (best performance): (1) a 5-repetition chair stand without the use of arms, (2) a progressive balance test, and (3) a 3-m usual walking speed measure (all in seconds).^23^

### Statistical Analysis

We described study participants according to sex, age, rheumatological diagnosis, anthropometric measures, and details of prednisolone treatment. Continuous normally distributed data were expressed as means and SDs, and nonnormally distributed data as medians and IQRs or ranges where appropriate. Normality was assessed using quantile-quantile plots and histograms. Associations between an AddiQoL-30 score of 85 or lower and the following factors were examined by crude prevalence ratios (PR) using binomial regression with robust variance estimator: basal cortisol levels, cortisol sample time, female sex, time since prednisolone cessation, body fat percentage, physical performance, age, Charlson Comorbidity Index, prednisolone starting dose and 6-month cumulative prednisolone exposure, treatment length, rheumatological diagnosis, and C-reactive protein, cholesterol, glycated hemoglobin, and hemoglobin levels.

Missing data for the questionnaire items were imputed using multiple imputation with predictive mean matching. The amount of missing responses were less than 3% in all but item 10 in AddiQoL-30 (“I am satisfied with my sex life”), with 9% missing responses. Internal consistency of questionnaires was determined using Cronbach α and McDonald ω.

Correlation between plasma cortisol and AddiQoL-30 and between morning cortisol and stimulated cortisol was examined using crude and adjusted linear regression after checking diagnostic plots of the residuals. Covariates in the adjusted model included sex, age, sample time, body fat percentage and handgrip strength, or sex and age for the morning cortisol model. Covariates were chosen based on observed prevalence ratios and theoretical connection. For comparisons between QoL measures, we employed a linear regression model with cluster-robust variance estimator to account for nonindependence between study sites. A 2-sided significance level of 5% was used for all models, and *P* values were accompanied by *q* values to account for multiple testing. These were calculated using the Benjamini-Hochberg procedure with a false discovery rate of .10. All statistical analyses were done using Stata version 17.0 SE (StataCorp).

## Results

**Figure 1.**
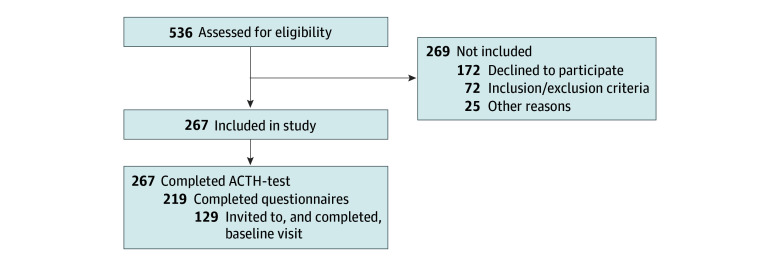
Flowchart of Study Inclusion Only a subset of participants were invited to baseline visit as the majority was allocated to a control group in the REPLACE study, which became full. ACTH indicates adrenocorticotropic hormone (corticotropin).

From March 2021 to March 2024, 536 patients were assessed for eligibility, of whom 267 (145 female [55%]; median [IQR] age, 73 [68 to 78] years) were enrolled and underwent an SST (Figure 1). Among these, 219 completed the questionnaires and 129 underwent additional investigations as part of a protocolled baseline visit. Of these, 90 (70%) had a diagnosis of PMR, 18 (14%) had a diagnosis of GCA, and 21 (16%) had both PMR and GCA. Pertinent characteristics of the participants are provided in Table 1.

**Table 1.** Characteristics of Study Participants

**Characteristic**	Participants, No. (%)
At screening (n = 267)	
Age, median (IQR), y	73 (68-78)
Sex	
Male	122 (45.7)
Female	145 (54.3)
At baseline (n = 129)	
Diagnosis	
PMR	90 (69.7)
GCA	18 (14.0)
Both	21 (16.2)
Time since diagnosis, median (IQR), y	3 (2-4)
Concomitant IL-6 inhibitor treatment	8 (6)
Prednisolone treatment	
Starting dose, median (IQR) [range], mg	15 (15-30) [5-75]
Treatment duration, median (IQR) [range], mo	13 (10-20) [3-180]
Cumulative dose, mean (SD), mg^a^	348 (209)
Anthropometrics	
BMI, median (IQR)^b^	27.5 (24.1-29.6)
Waist circumference, mean (SD), cm	96.2 (14.1)
Hip circumference, mean (SD), cm	105.7 (10.4)
Body fat percentage, mean (SD)	38.6 (7.9)
Clinical biochemistry	
Basal cortisol	
Mean (SD), nmol/L	295 (94)
Time of day of sample, median (IQR) [range], hh:mm	11:28 (10:08-12:44) [07:25-15:27]
Time between last prednisolone dose to cortisol measurement, median (IQR), d	39 (25-62)
C-reactive protein, median (IQR), mg/dL	0.3 (0.2-0.6)
Cholesterol	
Total, median (IQR), mg/dL	186 (155-216)
LDL, median (IQR), mg/dL	97 (77-127)
Triglycerides, median (IQR), mg/dL	97 (80-150)
HbA_1c_, mean (SD), %	5.6 (0.4)
Hemoglobin, mean (SD), g/dL	13.5 (1.3)

Abbreviations: BMI, body mass index; GCA, giant cell arteritis; HbA_1c_, glycated hemoglobin; IL-6, interleukin-6; LDL, low-density lipoprotein; PMR, polymyalgia rheumatica.

SI conversion factors: To convert cortisol to μg/dL divide by 27.588; C-reactive protein from mg/dL to mg/L, multiply by 10; HbA_1c_ from % of total hemoglobin to proportion of total hemoglobin, multiply by 0.01; hemoglobin from g/dL to g/L, multiply by 10.0; LDL from mg/dL to mmol/L, multiply by 0.0259; total cholesterol from mg/dL to mmol/L, multiply by 0.0259; triglycerides from mg/dL to mmol/L, multiply by 0.0113.

^a^ Cumulative dose in the 6 months leading up to baseline visit.

^b^ Calculated as weight in kilograms divided by height in meters squared.

### Prevalence of GIAI

Five of the 267 participants (1.9%; 95% CI, 0.8%-4.3%) exhibited a 30-minute cortisol level less than 420 nmol/L (366, 388, 400, 403, and 407 nmol/L, respectively) and by definition had biochemical GIAI. Their corresponding baseline cortisol levels were 191, 142, 217, 200, and 219 nmol/L. Median (IQR) time between last prednisolone dose and SST for all participants was 39 (25-62) days. Four out of 5 participants with GIAI had an AddiQoL-30 score of 85 or lower. The 4 participants (3 with GCA) with biochemical GIAI who underwent the baseline visit program were tested between 14 to 25 days after last prednisolone dose and after a treatment duration between 12 to 26 months. Baseline cortisol was sampled before 9 am in 22 participants where unadjusted linear regression showed an association between morning and stimulated cortisol levels with a coefficient of 0.65 (95% CI, 0.48-0.82) nmol/L stimulated cortisol per nmol/L morning cortisol (*R*^2^ = 0.47; *P* = .004; *q* = .03) (see eFigure in Supplement 1). Adjusting for sex and age, the coefficient was 0.57 (95% CI, 0.17-0.97; *R*^2^ = 0.61; *P* = .03; *q* = .07).

### Quality of Life

The mean (SD) AddiQoL-30 score (219 patients) was 89 (10), and 75 participants (34%; 95% CI, 28% to 41%) scored 85 or lower (symptomatic group) (Table 2). The symptomatic group scored lower in all 4 dimensions but more so as regards fatigue and musculoskeletal symptoms (Table 2; eTable 3 in Supplement 1). The internal consistency of AddiQoL-30 was good with a Cronbach α of 0.90 and a McDonald ω of 0.90. For CushingQoL, Cronbach α was 0.87 and McDonald ω was 0.88. Participants in the symptomatic group also scored worse on CushingQoL (mean [SD] score, 51 [16] vs 76 [13]; difference, 25; 95% CI, 16-34; *P* = .01; *q* = .05) (Table 2), and rated their overall sleep quality worse with a difference of 1.9 (95% CI, −0.1 to 4.0) points on a scale from 1 to 10 (mean [SD] score, 4.7 [2.1] vs 6.7 [2.0]; *P* = .05; *q* = .08) (Table 2).

**Table 2.** Quality of Life (QoL) Measures

Measure	All (N = 219)	AddiQoL ≤85 (symptomatic group) (n = 75)	AddiQoL >85 (asymptomatic group) (n = 144)	Absolute difference (95% CI)	Relative difference, % (95% CI)	*P* value	*q* value^a^
AddiQoL-30^b^							
Sum score total, mean (SD)	89 (10)	78 (7)	94 (6)	NA	NA	NA	NA
Sum score fatigue subscale, mean (SD)	22 (4)	18 (4)	24 (3)	6 (4-8)	33 (22-44)	.005	0.03
Sum score symptom subscale, mean (SD)	29 (4)	26 (3)	31 (2)	5 (4-6)	20 (15-25)	.004	0.03
Sum score emotion subscale, mean (SD)	24 (3)	22 (2)	25 (2)	3 (2-4)	15 (10-20)	.006	0.04
Sum score miscellaneous subscale, mean (SD)	14 (2)	12 (2)	15 (2)	2.6 (1.6-3.5)	21 (12-30)	.008	0.05
CushingQoL^c^							
Standardized score, mean (SD)	68 (18)	51 (16)	76 (13)	25 (16-34)	49 (31-67)	.007	0.05
Physical sub score, mean (SD)	58 (22)	47 (20)	64 (21)	17 (14-21)	36 (30-45)	.002	0.02
Psychosocial sub score, mean (SD)	71 (20)	53 (17)	80 (13)	27 (16-39)	51 (30-74)	.009	0.05
Single Item Sleep Quality Scale^d^							
Score, mean (SD)	6.0 (2.2)	4.7 (2.1)	6.7 (2.0)	1.9 (0.0-3.9)	43 (0-83)	.05	0.08

Abbreviations: AddiQoL-30, Addison’s disease-specific quality of life questionnaire; NA, not applicable.

^a^ *q* Values are reported using the Benjamini-Hochberg procedure with a false discovery rate of .10.

^b^ AddiQoL-30 score: scored in arbitrary units from 30 to 120 with lower score signifying more symptoms of adrenal insufficiency.

^c^ CushingQoL: scored from 1 to 100 with lower scores signifying more symptoms related to Cushing syndrome.

^d^ Single Item Sleep Quality Scale: Scored from 1 to 10 with lower scores indicating worse overall sleep quality.

### Variables Associated With AddiQoL-30 Score of 85 or Lower

Patients in the symptomatic group had lower basal cortisol levels compared with the asymptomatic group (263 nmol/L; 95% CI, 242-283 nmol/L vs 309 nmol/L; 95% CI, 295-324 nmol/L; *P* < .001; *q* = .009). The stimulated cortisol levels did not differ between the 2 groups (629 nmol/L; 95% CI, 600-657 vs 650 nmol/L; 95% CI, 632-668 nmol/L; *P* = .19; *q* = .09).

**Figure 2.**
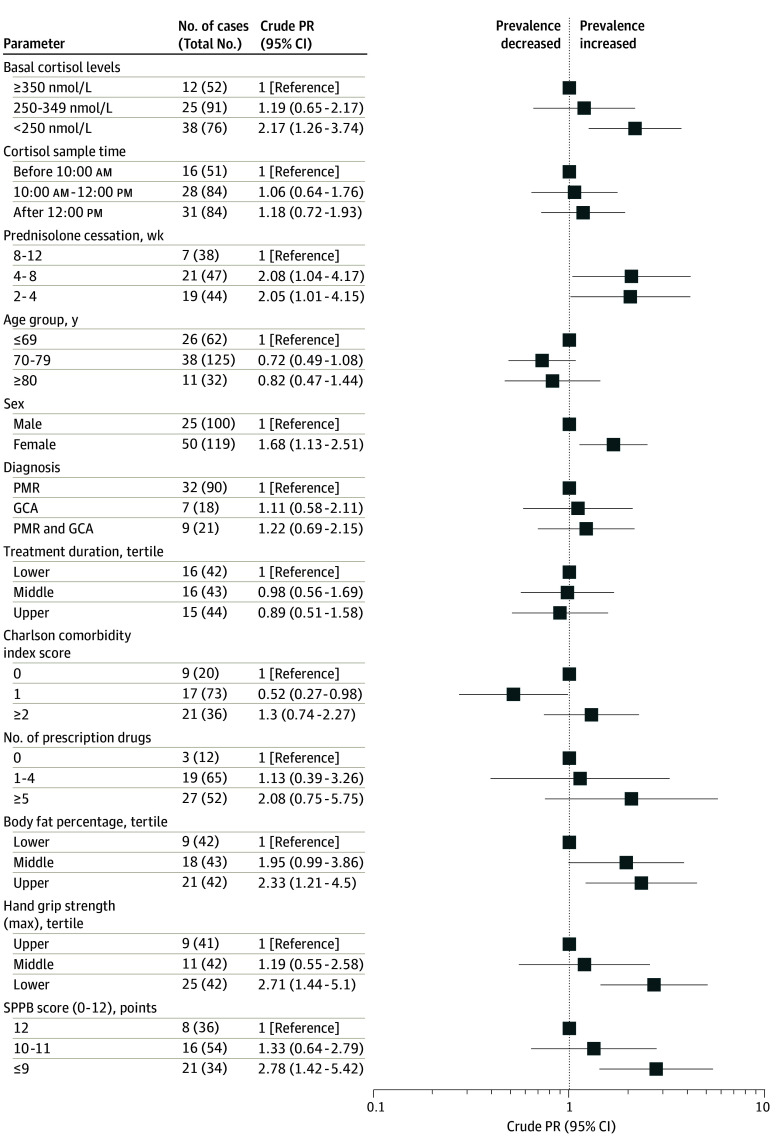
Crude Prevalence Ratios (PRs) for Associations of Variables With Increased Likelihood of AddiQoL-30 Score 85 or Lower Prednisolone cessation: time between last prednisolone dose and the corticotropin test. Charlson comorbidity index is scored without age. SI conversion factor: To convert cortisol to μg/dL divide by 27.588. GCA indicates giant cell arteritis; PMR, polymyalgia rheumatica; SPPB, Short Physical Performance Battery score.

Figure 2 shows crude prevalence ratios (PR) for a selected subset of variables associated with a score of 85 or lower, including low basal cortisol, female sex (PR, 1.68; 95% CI, 1.13-2.51), short duration of prednisolone cessation (PR, 2.05; 95% CI, 1.01-4.15), high body fat percentage (PR, 2.33; 95% CI, 1.21-4.50), reduced handgrip strength (PR, 2.71; 95% CI, 1.44-5.10), and lower physical performance (PR, 2.78; 95% CI, 1.42-5.42). Neither prednisolone starting dose nor 6-month cumulative prednisolone exposure, C-reactive protein, cholesterol, glycated hemoglobin, or hemoglobin were associated with a score of 85 or lower.

**Figure 3.**
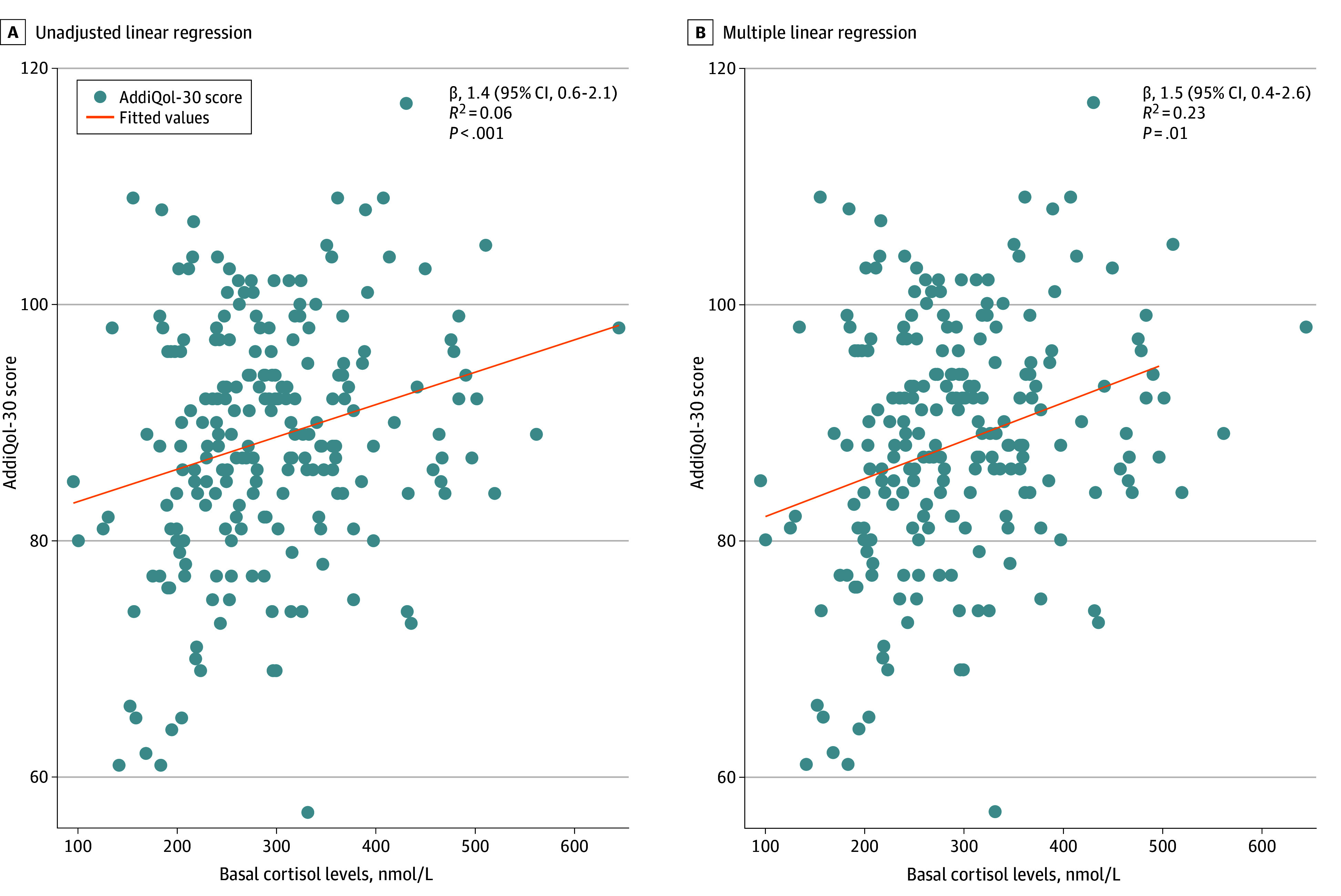
Linear Regression Models of Basal Cortisol and Addison Disease-Specific Quality of Life Questionnaire (Addiqol-30) Score A, Unadjusted linear regression. B, Multiple linear regression, adjusted for age, sex, body fat percentage, hand grip strength, and corticotropin stimulation test sample time. β-value is the slope, calculated as the AddiQol-30 score per 50 nmol/L cortisol. Removing the participant with basal cortisol higher than 600 nmol/L did not change the outcome. SI conversion factor: To convert cortisol to μg/dL divide by 27.588.

Unadjusted linear regression revealed a linear correlation between basal cortisol levels and AddiQoL-30 score, indicating an improvement of 1.4 (95% CI, 0.6-2.1) points in AddiQoL-30 score per 50 nmol/L increase in basal cortisol (*R*^2^ = 0.06; *P* < .001; *q* = .005) (Figure 3). The linear correlation remained after adjustment for sex, age, sample time point, handgrip strength and body fat percentage (1.5; 95% CI, 0.4 to 2.6) per 50 nmol/L cortisol (*R*^2^ = 0.23; *P* = .01; *q* = .07)] (Figure 3).

## Discussion

The prevalence of GIAI defined by the classic SST in our study cohort was very low as compared with previous reports.^10,24^ Notwithstanding this, 34% of the patients exhibited symptoms of AI comparable with patients with endogenous AI on stable hydrocortisone replacement therapy (eTable 3 in Supplement 1). Participants in the symptomatic group also had significantly lower basal cortisol levels, and we found a positive linear correlation between basal cortisol levels and a better AddiQoL-30 score.

The cutoff level of the SST corresponds to the 2.5th percentile in a healthy population, based on which one would expect 6 out of 267 individuals with normal adrenal function (as compared with the observed 5) to exhibit a stimulated plasma cortisol level less than 420 nmol/L.^25,26^ A meta-analysis^10^ from 2016 reported an overall GIAI prevalence after oral GC treatment of 49% where the majority of studies tested adrenal function during ongoing GC treatment, typically within 1 day after pausing GC treatment. A recent retrospective study^27^ in 206 patients referred with suspected AI reported an overall GIAI prevalence of 33.5% based on an SST while on low dose prednisolone treatment. However, when retesting during further dose tapering, most patients recovered HPA function with only 1.7% failing 3 or more SSTs. We tested all eligible patients regardless of clinical suspicion, and performed the SST 2 to 12 weeks after planned prednisolone cessation preceded by tapering of the dose including approximately 20 weeks on 5 or less mg/d.

To our knowledge, this study is the first to provide concomitant data on biochemical and clinical adrenal function from a protocolled trial with rigorous inclusion and exclusion criteria. Among the patients experiencing more AI symptoms, we observed a higher frequency of women, a higher body fat percentage, and a lower handgrip strength and physical performance. We did not find associations between AI symptom burden and age, comorbidity, or underlying diagnosis, all of which could have been confounders for a reduced AddiQoL-30 score. However, our limited sample size does not allow us to draw firm conclusions in this regard. Interestingly, the group with AI symptoms also exhibited lower basal cortisol levels, which could suggest a subtle suppression of adrenocortical function not captured by the SST, which provides a supraphysiological stimulus. After tapering and cessation of pharmacological GC treatment, a so-called steroid withdrawal syndrome may follow, which consists of physical and/or psychological dependence upon GC and which may persist despite recovery of adrenal function as judged by the SST and absence of relapse of the underlying disease.^15,16,28^ Our findings extend and support the existence and significance of this syndrome, and it is evident that it is negatively associated with patient-perceived QoL and may lead to prolonged prednisolone treatment and adverse effects associated herewith.^1,29^ A better understanding of the syndrome based on data within the framework of protocolled randomized clinical trials is needed to reduce the complications of long-term GC therapy.

Our findings suggest that a 250 μg SST after planned cessation of prednisolone treatment is not cost effective as a routine follow up and could be restricted to patients with severe symptoms, which supports and strengthens the recommendations from a recently published guideline.^28^ This guideline also recommends the use of morning cortisol for assessing adrenal function with levels between 150 to 300 nmol/L a gray area. We collected morning cortisol (7 am to 9 am) from 22 patients, 7 of whom had cortisol levels between 150 to 300 nmol/L, all with a normal SST.

As mentioned, the SST provides a supraphysiological stimulus, and, to our knowledge, it has not been compared with features of endogenous cortisol secretion, such as production rate and circadian or ultradian rhythmicity or to symptoms and signs of AI.^10,30^ In this context, there is a need for additional biochemical tools to measure GC status during and after pharmacological GC treatment, but such a method must be tested rigorously in a clinical trial.^31^ A potential candidate for further scrutiny could be a recently published bioassay for quantification of glucocorticoid activity in serum.^32^ However, as it stands, the SST remains the criterion standard for diagnosing AI.

### Strengths and Limitations

The strengths of our study include the rigorous and protocolled design within the framework of a randomized clinical trial and the fact that we invited all eligible patients with PMR and/or GCA in a nationwide study using state of the art cortisol assays. Our study did not capture patients unable to sustain prednisolone cessation of at least 2 weeks, which is a limitation that could have affected the prevalence of AI. Furthermore, nearly one third of the invitees declined participation, which could have introduced selection bias. Lastly, our definition of symptomatic AI is based on a questionnaire developed to detect symptoms in patients with primary AI.

## Conclusions

In this study, the prevalence of GIAI 2 or more weeks after cessation of prednisolone treatment in patients with PMR and GCA was very low (1.9%) assessed by the 250 μg SST. One-third of the patients exhibited symptoms compatible with AI, despite a normal response to the SST. These patients had significantly lower basal cortisol levels compared with patients without symptoms. The latter findings suggest an unmet need for additional diagnostic and therapeutic tools to aid health care professionals involved in the management of patients receiving glucocorticoid treatment including the glucocorticoid withdrawal syndrome. Future studies in this field should include randomized clinical trials with both physician- and patient-reported outcomes.

## Notes

Supplement 1. **eTable 1. **Eligibility Criteria **eTable 2.** Overview of Studies Using AddiQoL-30 **eTable 3.** Mean AddiQoL-30 Score by Question and Dimension **eFigure.** Univariate and Multivariate Linear Regressions of Basal and Stimulated Cortisol Values

Supplement 2.
Data Sharing Statement
