# Supplementary material for: Changes in Adrenal Function and Insufficiency Symptoms After Cessation of Prednisolone
Source: JAMA Netw Open. 2025 Mar 18;8(3):e251029. doi: 10.1001/jamanetworkopen.2025.1029 (PMC11920843; doi:10.1001/jamanetworkopen.2025.1029)
Supplement: Supplement 1. — eTable 1. Eligibility Criteria eTable 2. Overview of Studies Using AddiQoL-30 eTable 3. Mean AddiQoL-30 Score by Question and Dimension eFigure. Univariate and Multivariate Linear Regressions of Basal and Stimulated Cortisol Values [file jamanetwopen-e251029-s001.pdf]

Supplemental Online Content

Hansen SB, Dreyer AF, Jørgensen NT, et al. Adrenal function and insufficiency symptoms after cessation of prednisolone. *JAMA Netw Open*. 2025;8(3):e251029. doi:10.1001/jamanetworkopen.2025.1029

eTable 1. Eligibility Criteria

eTable 2. Overview of Studies Using AddiQoL-30

eTable 3. Mean AddiQoL-30 Score by Question and Dimension

eFigure. Univariate and Multivariate Linear Regressions of Basal and Stimulated Cortisol Values

This supplemental material has been provided by the authors to give readers additional information about their work.

© 2025 Hansen SB et al. *JAMA Network Open*.

eTable 1. Eligibility Criteria

Inclusion criteria

- Age ≥ 50 years
- Polymyalgia rheumatica and/or giant cell arteritis
- Prednisolone treatment for ≥ 12 weeks
- Prednisolone treatment ceased 2-12 weeks prior to inclusion

Exclusion criteria

- Cushing syndrome of endogenous origin
- Primary or secondary adrenal insufficiency
- Severe comorbidity
  - Severe heart disease (New York Heart Association NYHA class IV)
  - Kidney disease (Chronic Kidney Disease CKD stage 4, eGFR <30ml/min)
  - Liver cirrhosis
  - Active cancer
  - Known severe immunodeficiency
- Alcohol consumption > 21 units per week
- Planned major surgery during the study period at study entry
- Use of drugs that interfere with cortisol metabolism/measurements:
  - Systemic estrogen treatment (discontinued < 1 month before inclusion)
  - Strong inducers or inhibitors of Cytochrome P450 3A4 (CYP3A4)
- Corticosteroid formulations:
  - Intra-articular or intra-muscular injections discontinued less than 60 days before inclusion
  - Topical: European steroid group IV-V used in the genital area
- The following corticosteroids formulations were permitted during the study period:
  - Inhaled (paused ≥ 7 days before corticotropin test)
  - Topical: European steroid group I-III, and European steroid group IV-V used in the non-genital area only (paused ≥ 2 days before corticotropin test)
  - Nasal spray, eye/ear-drops (paused ≥ 2 days before corticotropin test)

© 2025 Hansen SB et al. *JAMA Network Open*.

eTable 2. Overview of Studies Using AddiQoL-30 with reporting method as described in Oksnes et al.[1] Mean raw and weighted AddiQoL-30 score is listed for PAI + SAI and healthy participants. AI, adrenal insufficiency; PAI, primary adrenal insufficiency; SAI, secondary adrenal insufficiency.

Study	Size of study - n	Type of AI	Mean baseline AddiQoL-30 score	Weight based on study size	Weight*average
Oksnes et al 2012[1]	99	PAI	89	0·169	15·1
Quinkler et al 2015[2]	30	PAI, SAI	83·8	0·051	4·3
Quinkler et al 2015[2]	20	PAI, SAI	84	0·034	2·9
Oksnes et al 2014[3]	33	PAI	86·9	0·056	4·9
Meyer et al 2018[4]	110	PAI	88	0·188	16·5
Gagliardi et al 2014[5]	10	PAI	99	0·017	1·7
Isidori et al 2018[6]	89	PAI, SAI	82	0·152	12·5
Chihaoui et al 2020[7]	50	SAI	81·2	0·085	6·9
Giordano et al 2016[8]	19	PAI	76	0·032	2·5
Gasco et al 2023[9]	21	SAI	86	0·036	3·1
Didriksen et al 2021[10]	72	PAI	83	0·123	10·2
Zdrojowy-Wełna et al 2023[11]	32	PAI	85	0·055	4·6
Sum (AI patients)	585	PAI, SAI	85·3	1	85·2
Oksnes et al 2012[1]	462	Healthy	97	1	97

© 2025 Hansen SB et al. *JAMA Network Open*.

eTable 3. Mean AddiQoL-30 Score by Question and Dimension. Larger positive difference means poorer QoL in the symptomatic group compared to the asymptomatic group. Each question has a possible range of 1 to 4. QoL, quality of life; NA, not applicable.

Question	Dimension	Wording	Entire group N = 220		Symptomatic group ≤85 N = 75		Asymptomatic group >85 N = 145		Difference between means
			Mean	SD	Mean	SD	Mean	SD	
Total score	Na	Na	88·6	10·3	77·6	6·8	94·3	6·3	16·7
Question 1	Fatigue	I feel good about my health	2·7	0·7	2·3	0·6	2·9	0·6	0·7
Question 2	Fatigue	I can keep going during the day without feeling tired	2·5	0·8	1·9	0·7	2·8	0·6	0·8
Question 3	Fatigue	Normal daily activities make me tired	3·0	0·6	2·7	0·7	3·2	0·5	0·5
Question 4	Fatigue	I have to struggle to finish jobs	3·1	0·7	2·7	0·7	3·3	0·6	0·7
Question 5	Fatigue	I have to push myself to do things	3·1	0·7	2·6	0·7	3·4	0·6	0·8
Question 6	Symptom	I lose track of what I want to say	3·4	0·6	3·1	0·6	3·6	0·5	0·5
Question 7	Miscellaneous	I sleep well	2·6	0·7	2·3	0·6	2·8	0·7	0·5
Question 8	Miscellaneous	I feel rested when I wake up in the morning	2·5	0·7	2·1	0·7	2·7	0·7	0·7
Question 9	Symptom	I feel unwell first thing in the morning	3·3	0·7	2·8	0·7	3·5	0·6	0·8
Question 10	Miscellaneous	I am satisfied with my sex life	2·4	1·0	2·0	0·9	2·6	1·0	0·6
Question 11	Emotion	I am relaxed	2·7	0·6	2·3	0·5	2·9	0·6	0·6
Question 12	Emotion	I feel low or depressed	3·5	0·6	3·0	0·6	3·7	0·5	0·7
Question 13	Emotion	I am irritable	3·4	0·6	3·0	0·5	3·6	0·5	0·5
Question 14	Emotion	I find it difficult to think clearly	3·5	0·6	3·1	0·7	3·7	0·5	0·7
Question 15	Emotion	I feel lightheaded	3·5	0·7	3·2	0·7	3·6	0·6	0·4
Question 16	Symptom	I sweat for no particular reason	3·5	0·7	3·3	0·8	3·7	0·5	0·4
Question 17	Symptom	I get headaches	3·5	0·6	3·2	0·6	3·6	0·5	0·4
Question 18	Symptom	I get nauseous	3·7	0·5	3·5	0·6	3·9	0·4	0·3
Question 19	Symptom	My joints and/or muscles ache	2·5	0·7	2·0	0·7	2·7	0·6	0·7
Question 20	Symptom	I have back pain	3·0	0·8	2·7	0·8	3·2	0·7	0·5
Question 21	Symptom	My legs feel weak	2·9	0·8	2·3	0·7	3·1	0·6	0·8
Question 22	Symptom	I worry about my health	3·1	0·7	2·7	0·6	3·3	0·6	0·6
Question 23	Fatigue	My ability to work is limited	2·9	0·7	2·4	0·8	3·2	0·6	0·7
Question 24	Emotion	I can concentrate well	1·8	0·8	1·9	0·7	1·7	0·9	-0·2
Question 25	Emotion	I am happy	2·7	0·7	2·5	0·6	2·8	0·8	0·3
Question 26	Fatigue	I feel full of energy	2·2	0·7	1·6	0·6	2·5	0·6	0·8
Question 27	Fatigue	I feel physically fit	2·4	0·7	1·9	0·8	2·6	0·5	0·7
Question 28	Miscellaneous	I get ill more easily than others	3·4	0·7	3·1	0·8	3·5	0·6	0·4
Question 29	Miscellaneous	I take a long time to recover from illnesses	3·1	0·7	2·8	0·8	3·3	0·7	0·5
Question 30	Emotion	I cope well in emotional situations	2·8	0·6	2·6	0·6	2·9	0·6	0·3
Fatigue	mean	Na	2·7	0·7	2·3	0·7	3·0	0·6	0·7
Symptom	mean	Na	3·2	0·7	2·8	0·7	3·4	0·6	0·6
Miscellaneous	mean	Na	2·8	0·8	2·5	0·7	3·0	0·7	0·5
Emotion	mean	Na	3·0	0·7	2·7	0·6	3·1	0·6	0·4
All dimensions	mean	Na	3·0	0·7	2·6	0·7	3·1	0·6	0·6

© 2025 Hansen SB et al. *JAMA Network Open*.

eFigure. Univariate and Multivariate Linear Regressions of Basal and Stimulated Cortisol Values. A) Univariate linear regression on subgroup with basal values taken ≤ 9 am, B) Multivariate linear regression on subgroup with basal values taken ≤ 9 am adjusted for sex and age, C) Univariate linear regression of all 267 participants, D) Multivariate linear regression of all 267 participants adjusted for sex and age.

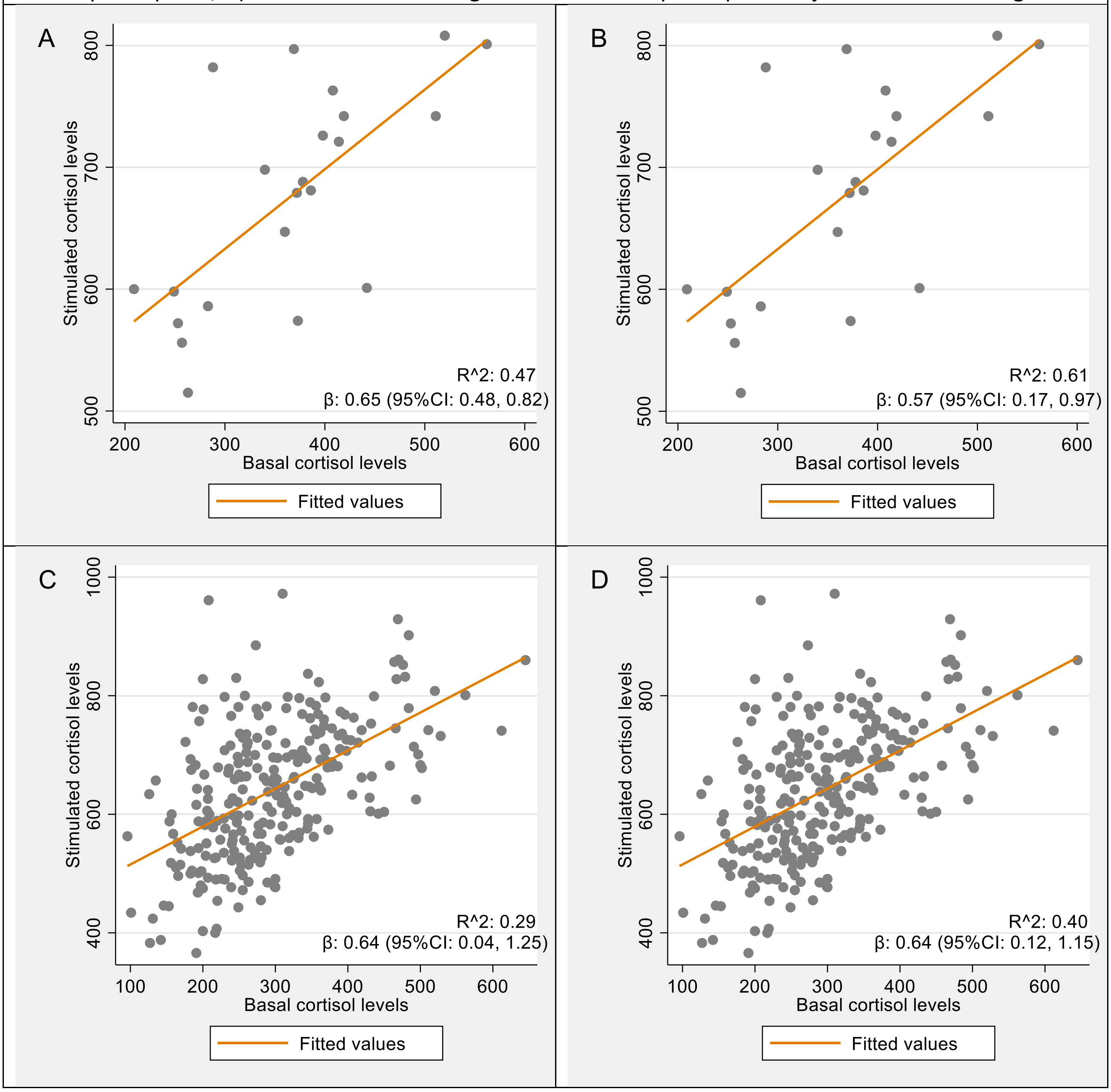

© 2025 Hansen SB et al. *JAMA Network Open*.

eReferences

1. Oksnes M, Bensing S, Hulting AL, et al. Quality of life in European patients with Addison's disease: validity of the disease-specific questionnaire AddiQoL. *The Journal of clinical endocrinology and metabolism.* 2012;97(2):568-576.
2. Quinkler M, Miodini Nilsen R, Zopf K, Ventz M, Oksnes M. Modified-release hydrocortisone decreases BMI and HbA1c in patients with primary and secondary adrenal insufficiency. *European journal of endocrinology.* 2015;172(5):619-626.
3. Oksnes M, Björnsdottir S, Isaksson M, et al. Continuous subcutaneous hydrocortisone infusion versus oral hydrocortisone replacement for treatment of addison's disease: a randomized clinical trial. *J Clin Endocrinol Metab.* 2014;99(5):1665-1674.
4. Meyer G, Koch M, Herrmann E, Bojunga J, Badenhoop K. Longitudinal AddiQoL scores may identify higher risk for adrenal crises in Addison's disease. *Endocrine.* 2018;60(2):355-361.
5. Gagliardi L, Nenke MA, Thynne TR, et al. Continuous subcutaneous hydrocortisone infusion therapy in Addison's disease: a randomized, placebo-controlled clinical trial. *The Journal of clinical endocrinology and metabolism.* 2014;99(11):4149-4157.
6. Isidori AM, Venneri MA, Graziadio C, et al. Effect of once-daily, modified-release hydrocortisone versus standard glucocorticoid therapy on metabolism and innate immunity in patients with adrenal insufficiency (DREAM): a single-blind, randomised controlled trial. *Lancet Diabetes Endocrinol.* 2018;6(3):173-185.
7. Chihaoui M, Mimita W, Oueslati I, et al. Prednisolone or hydrocortisone replacement in patients with corticotrope deficiency fasting during Ramadan result in similar risks of complications and quality of life: a randomized double-blind controlled trial. *Endocrine.* 2020;67(1):155-160.
8. Giordano R, Marzotti S, Balbo M, et al. Metabolic and cardiovascular profile in patients with Addison's disease under conventional glucocorticoid replacement. *J Endocrinol Invest.* 2009;32(11):917-923.
9. Gasco V, Giannelli J, Campioni L, et al. Benefits of dual-release hydrocortisone treatment on central adiposity and health-related quality of life in secondary adrenal insufficiency. *J Endocrinol Invest.* 2023;46(3):587-597.
10. Didriksen NM, Sævik Å B, Sortland LS, Øksnes M, Husebye ES. Sex-Specific Limitations in Physical Health in Primary Adrenal Insufficiency. *Front Endocrinol (Lausanne).* 2021;12:718660.
11. Zdrojowy-Wełna A, Stańska A, Halupczok-Żyła J, Szcześniak D, Bolanowski M. Health-Related Quality of Life in Patients with Primary Adrenal Insufficiency. *J Clin Med.* 2023;12(23).

© 2025 Hansen SB et al. *JAMA Network Open*.
