# Supplementary material for: Changes in Adrenal Function and Insufficiency Symptoms After Cessation of Prednisolone
Source: JAMA Netw Open. 2025 Mar 18;8(3):e251029. doi: 10.1001/jamanetworkopen.2025.1029 (PMC11920843; doi:10.1001/jamanetworkopen.2025.1029)
Supplement: Supplement 2. — Data Sharing Statement [file jamanetwopen-e251029-s002.pdf]

Data Sharing Statement

Hansen. Adrenal Function and Insufficiency Symptoms After Cessation of Prednisolone. *JAMA Netw Open*. Published March 18, 2025. doi:10.1001/jamanetworkopen.2025.1029

Data
Data available: No

Additional Information
Explanation for why data not available: Deidentified participant data and Study protocol, and analytic code are available upon reasonable request.
